# Neural correlates of thermal stimulation during active touch

**DOI:** 10.3389/fnins.2023.1320417

**Published:** 2024-01-08

**Authors:** Wanjoo Park, Georgios Korres, Muhammad Hassan Jamil, Mohamad Eid

**Affiliations:** Engineering Division, New York University Abu Dhabi, Saadiyat Island, Abu Dhabi, United Arab Emirates

**Keywords:** thermal sensation, active touch, EEG response, human-computer interaction, power spectral density

## Abstract

**Introduction:**

Thermal feedback technologies have been explored in human-computer interaction to provide secondary information and enhance the overall user experience. Unlike fast-response haptic modalities such as vibration and force feedback, the human brain's processes associated with thermal feedback are not fully understood.

**Methods:**

In this study, we utilize electroencephalography (EEG) brain imaging to systematically examine the neural correlates associated with a wide range of thermal stimuli, including 9, 15, 32, and 42°C, during active touch at the fingertip. A custom experimental setup is developed to provide thermal stimulation at the desirable temperature levels. A total of 30 participants are recruited to experience the four levels of thermal stimulation by actively touching a thermal stimulation unit with the index finger while recording brain activities via EEG. Time-frequency analysis and power spectral density (PSD) of the EEG data are utilized to analyze the delta, theta, alpha, beta, and gamma frequency bands.

**Results:**

The results show that the delta, theta, and alpha PSDs of 9 and 15°C stimuli are significantly higher than the PSDs of 32 and 42°C in the right frontal area during the early stage of the stimulation, from 282 ms up to 1,108 ms (One-way ANOVA test, Holm-Bonferroni correction, *p* < 0.05). No significant differences in PSDs are found between 9 and 15°C thermal stimuli or between 32 and 42°C thermal stimuli.

**Discussion:**

The findings of this study inform the development of thermal feedback system in human-computer interaction.

## 1 Introduction

Haptic technologies have primarily focused on fast-response modalities such as vibration and force feedback due to their promising potential to convey real-time information to the user (Dangxiao et al., 2019). On the contrary, slow-response modalities such as thermal feedback have not been thoroughly investigated due to slow-response characteristics and challenges in thermal actuation (control, power consumption, wearability, etc.) (Jones, 2016). Thermal feedback has been used to convey thermally encoded information in environments in which vibrotactile or force feedback might be masked by noise and/or movement (Wilson et al., 2013). Moreover, several studies reported how thermal stimulation can be used to modulate other tactile sensations. For instance, thermal feedback can improve object recognition when visual cues are limited (Wilson et al., 2012) and produce a more realistic feeling in teleoperation or virtual reality (Fermoselle et al., 2022). Thermal stimulation is also highly correlated with influencing emotional responses (Wilson et al., 2016).

Limited psychophysical research has been conducted to characterize the human haptic processing of thermal sensations. The thermal experience is principally dependent on the thermal exchange between the skin and the touched object, which triggers responses from thermal receptors. The perceived temperature is determined by several variables (Ho and Jones, 2006), including the initial temperature of the skin and the touched object, the thermal properties of the material (heat capacity and conductance), body part and contact area, the ambient temperature, and even the individual experience of the user (Vidyarini and Maeda, 2019). Therefore, evaluating the perceptual experience of thermal stimulation is a very challenging task. An interesting approach involves examining the neural correlates to evaluate the user experience of thermal stimulation.

Despite the significant number of studies on the perception of thermal stimulation, understanding the neural processes associated with thermal stimulation is still very challenging (Tayeb et al., 2022). Over the last decade, the quantification of various haptic sensations using neuroimaging techniques has been widely studied and investigated (Alsuradi et al., 2020a). Researchers have been relying on various neuroimaging techniques to evaluate the human haptic experience, including functional magnetic resonance imaging (fMRI) (Karim and Likova, 2018), positron emission tomography (PET) (McGlone et al., 2012), functional near-infrared spectroscopy (fNIRS), and electroencephalography (EEG). EEG can readily have a superior temporal resolution, is compatible with haptic devices, and offers a relatively lower cost (Alsuradi et al., 2020a). The EEG-based approach has been used to evaluate haptic experiences in several settings, such as the detection of tactile feedback on a touchscreen device (Alsuradi et al., 2020b), the identification of the task type (active vs. passive) (Miura et al., 2014), texture classification (Eldeeb et al., 2020), pain perception (Tu et al., 2016), and grasping task identification (Cisotto et al., 2018).

Wang et al. (2021) studied changes in EEG rhythms associated with thermal stimulation in the head at 33–41°C, induced through a laser, and observed a decrease in EEG power topographic patterns source. An et al. (2018) used magnetoencephalography to study thermal stimulation with a laser. Results demonstrated suppression of the alpha and beta band power in the bilateral sensorimotor cortex and delta band power increase in the frontal, temporal, and cingulate cortices. An fMRI study proposed a support vector machine (SVM) model to discriminate between painful and non-painful thermal stimulation, achieving an accuracy of 81% (Brown et al., 2011). Another study focused on the power modulation of different oscillatory components and their sensitivity to thermal comfort variations using EEG recordings (Breton et al., 2019). The study demonstrated a direct modulation of EEG in different frequency bands in accordance with the thermal conditions, as well as a direct correlation with thermal comfort modulations. A recent study examined spatial, temporal, and spectral patterns of brain responses to different thermal stimulation ranging from very intense (extremely cold and hot stimuli), intense (moderately cold and hot stimuli), to innocuous (a warm stimulus) (Tayeb et al., 2022). Results demonstrated that very intense thermal stimuli elicit a decrease in alpha power compared to intense and innocuous stimulation. Furthermore, spatio-temporal analysis reveals that in the first 400 ms post-stimulus, brain activity increases in the prefrontal and central brain areas for very intense stimulation, whereas for intense stimulation, high activity of the parietal area was observed post-500 ms. Chang et al. (2005) examined the EEG data during and after thermal stimulation of the left hand with warm and cold thermal stimulation and showed a significant increase in theta and alpha bands after cold stimulation compared to the baseline. Lv et al. (2017) conducted a study in which they delivered warm temperature stimuli to two different media: a metal thermostat and thermostated water. Results suggested that the neural responses in different EEG frequency bands (delta, theta, and beta) were sensitive to different influence factors (such as the ambient temperature) during local hand thermal stimulation.

In this study, we utilize EEG brain imaging to systematically examine the neural correlates associated with a wide range of thermal stimulation, including 9°C, 15°C, 32°C, and 42°C-, thermoreceptors respond over a temperature range of 5–45°C (Darian-Smith and Johnson, 1977). To that end, an experimental instrument capable of rendering the four levels of temperatures on a thermal pad with a ±1.5°C accuracy was developed. The participants perform tasks by actively reaching out to the thermal pad and feeling the thermal stimulation with the fingertip of the right hand. The ultimate goal of this study is to develop a neural means that provides a quantitative, real-time, and non-intrusive evaluation of the user experience with thermal stimulation. Our hypothesis is that there will be differences in certain frequency bands and/or brain areas based on the thermal sensation of touching objects of different temperatures with the fingertips.

## 2 Materials and methods

### 2.1 Participants

A total of 30 healthy adults participated in this study. These included 21 males, 8 females, and 1 unidentified, age range 18–50 years old. Inclusion criteria are: adults aged 18 years and older and right-handed. Exclusion criteria include participants below the age of 18 or left-handed. All participants were healthy, with no prior physical or neuropsychiatric illness as confirmed through self-reporting. We considered whether a physical illness would cause hand and arm movements and whether a neuropsychiatric illness would cause a person to have unusual neural signaling. The study was conducted in full compliance with the ethical standards outlined in the Declaration of Helsinki, following its guidelines and regulations, and after obtaining approval from New York University Abu Dhabi Institutional Review Board (IRB: #HRPP–2020–80). Each participant signed an informed consent form in accordance with the IRB ethics. All participants received monetary compensation at the end of the experimental session. No identifying information was collected from the participants.

### 2.2 Experimental setup

**Figure 1:**
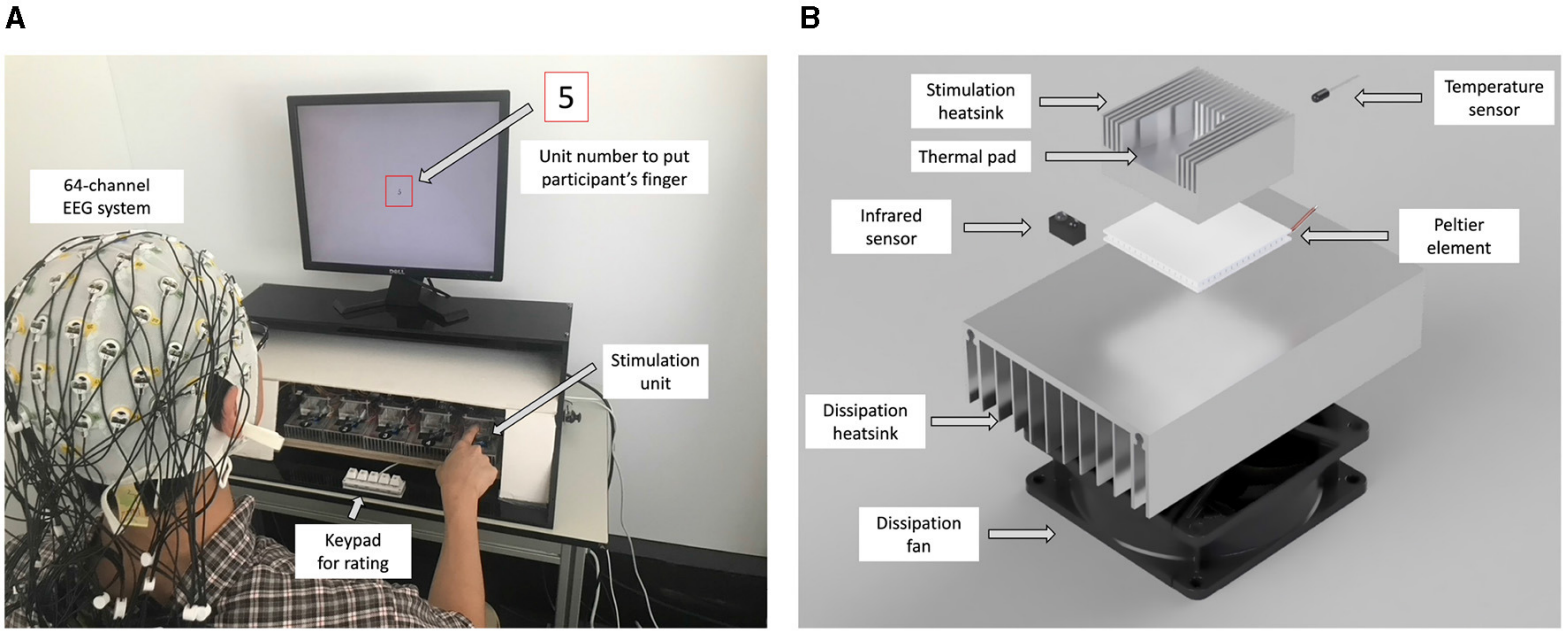
The experimental setup and thermal stimulation unit assembly. **(A)** Experimental setup. **(B)** An exploded rendering of the thermal stimulation unit assembly.

**Figure 2:**
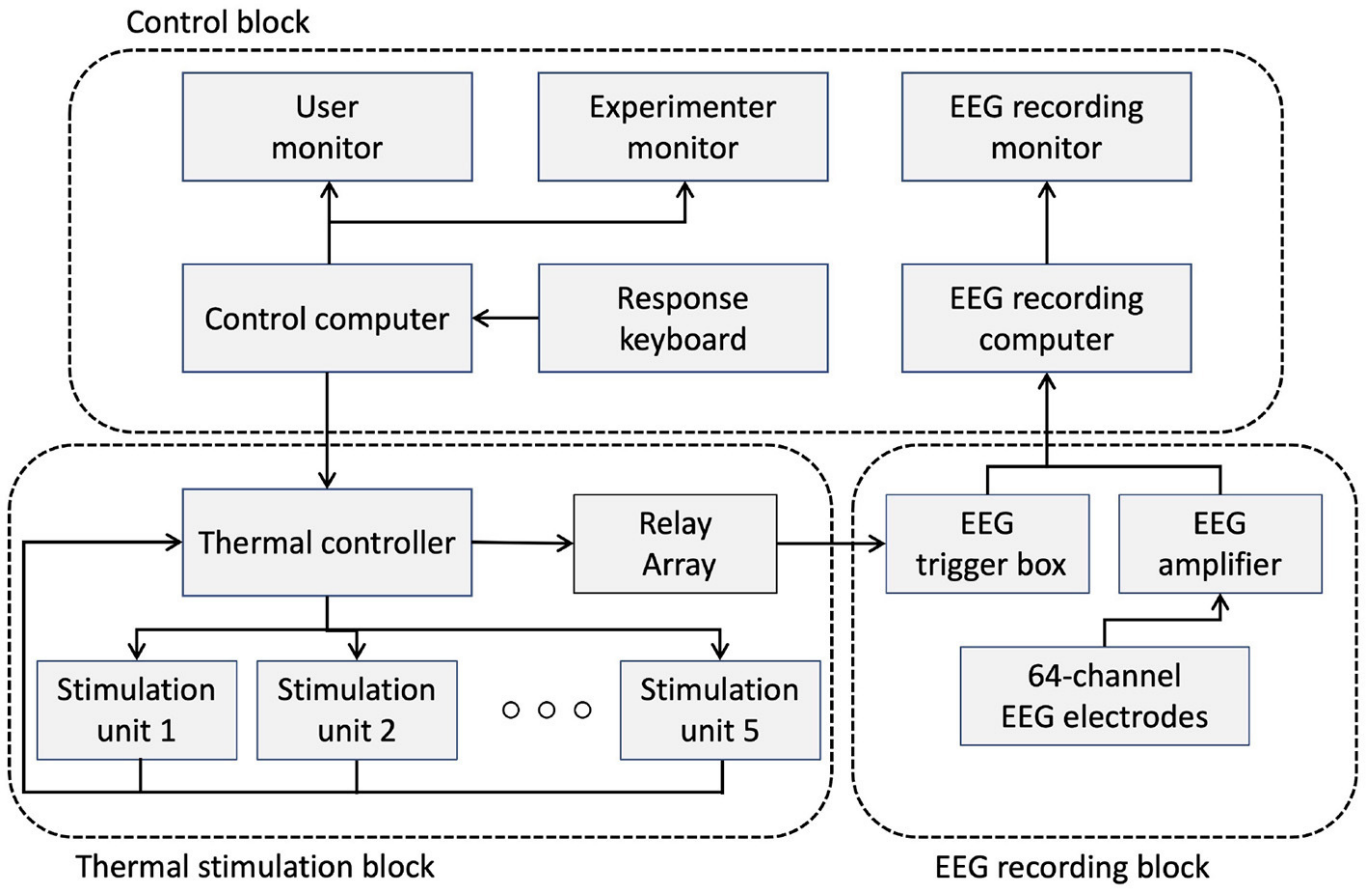
Schematic diagram of the experimental configuration.

A custom thermal display was developed to deliver different levels of thermal stimulation. Figure 1 shows the experimental setup and structure of the thermal stimulation unit. A schematic diagram describing how the setup works is shown in Figure 2. The experimental setup consisted of three blocks that managed the experimental protocol and the recording of EEG data: the control block, the thermal stimulation block, and the EEG recording block. The control block contains a desktop computer that was responsible for running the experimental protocol and recording the EEG data. The control block manages three monitors in the setup. The first was used for instructing the user through the experimental protocol while the thermal stimulation was provided. A second screen monitored the overall experiment status for the conductor of the experiment, while the third monitor was used to visualize the incoming EEG data as it was recorded. A 5-key keypad was connected to the control PC for the user to self-report the perceived thermal experience after every stimulation.

The thermal stimulation block was responsible for rendering five temperature levels (4 stimulation levels and one reference stimulation at 23°*C*) at the thermal pads. The reference temperature of 23°C is the ambient temperature in the laboratory environment. We chose 9°C and 42°C as the minimum and maximum temperatures that humans can experience without pain (Darian-Smith and Johnson, 1977), and we chose 15°C and 32°C between these two extremes and the ambient temperature. It consisted of 5 thermal stimulation units, labeled 1 to 5, where the labels were used to instruct the participant as to where to place their fingertip during the experiment. As shown in Figure 1B, each unit consisted of a Peltier element (TEC 12706) to provide the thermal actuation, two aluminum heatsinks mounted on both surfaces of the Peltier element, a DS18B20 temperature sensor to provide feedback about the actual temperature of the Peltier element, a thermal pad where the participant was supposed to touch through their fingertip, and an IR-proximity sensor (TCRT5000) to detect when the user's finger touches the thermal pad. The bottom side heatsink was equipped with a fan for active thermal dissipation, while on the upper side of the Peltier element, a custom heatsink was placed on which thermal dissipation fins were machined using a CNC mill to enhance dissipation and create a space for the user's fingertip. A 3 mm blind hole was also drilled on the upper heatsink to host the temperature sensor as close as possible to the thermal pad to monitor its temperature.

The rendered temperature was controlled using a BTS7960 H-Bridge module and a microcontroller (ATMEGA2560). The H-Bridge module was used to provide sufficient current to actuate the Peltier element. The microcontroller was responsible for switching and maintaining the desired temperature using a PID control loop. The microcontroller was also responsible for generating the trigger signals that were driving the relay module to set the appropriate triggers to the EEG trigger box. Finally, a relay was used to issue trigger signals to the EEG trigger box according to the temperature of the thermal stimulation. During the experiment, the ambient temperature was maintained at 23 ± 1 °C.

The EEG recording block represents the system responsible for recording the EEG data (an actiCHamp amplifier with the Brain Vision 80 Recorder Version 1.21.0201, Brainproducts GmbH, Germany). The system consisted of the EEG cap containing 64 active electrodes, the EEG amplifier with the DAQ unit, and the trigger box, which was responsible for labeling the onset of the stimulation (the time when the participant makes contact with the thermal pad through their fingertip) so that all EEG recordings were synchronized with the corresponding thermal simulations in accordance with the experiment protocol.

### 2.3 Experimental protocol

Participants were recruited by ads posted on the university campus. After completing the consent form, participants were briefed about the study and the experimental setup. Once the introduction was completed, a 64-electrode cap following the 10–20 international system was placed on the participant's scalp. The online reference electrode was positioned at the FCz location, while the ground electrode was positioned at the FPz location. The experimenter applied conductive gel to make sure that the input impedance on each electrode was kept below 15 kΩ for high-quality EEG recording.

**Figure 3:**
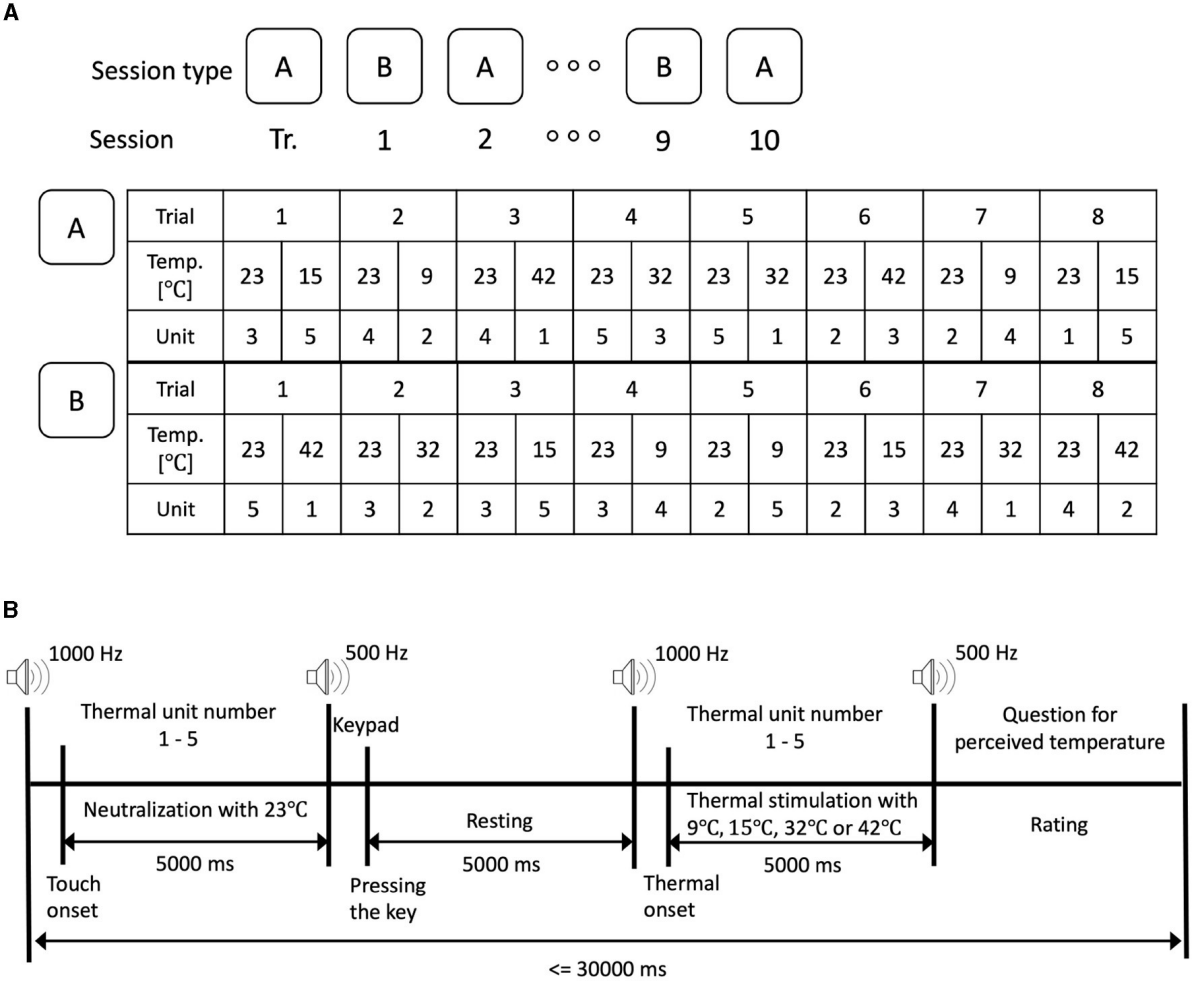
The experimental protocol. **(A)** Experimental protocol with two types of sessions. **(B)** Experimental protocol of a trial.

The protocol was designed in accordance with the purpose of the research and the capabilities/limitations of the thermal stimulation hardware. A long resting time was allowed to minimize the human thermal receptors saturation/fatigue. It also allowed sufficient time for temperature change in the thermal unit. Participants completed a training session to familiarize themselves with the experimental protocol and the temperature stimuli. Very cold and very hot temperature stimuli, namely 9 °C and 42 °*C*, were used in case the participants had not experienced extreme temperatures before the experiment. As illustrated in Figure 3, participants completed a training session followed by a total of 10 sessions that were divided into two types (A and B, shown in Figure 3A). The training session was introduced to provide sufficient acquaintance with the experimental setup and protocol. The training session had the same number of trials and followed the same protocol as the experiment sessions. The experiment sessions (A and B) were conducted with a counter-balanced sequence. Each session consisted of eight counter-balanced trials (using the four-by-four Latin Square order) where each trial was divided into neutralization and thermal stimulation periods. The neutralization period was introduced to normalize the initial thermal state that preceded the thermal stimulation.

A 1,000 Hz tone indicated the start of the trial. The stimulation unit number was displayed on the screen instructing the participant to touch the respective stimulation unit (units are numbered 1 to 5) which was set to neutralization period. After the thermal onset was detected indicating contact between the participant's fingertip and the thermal pad, 5,000 ms of neutralization period with 23 °C was experienced by the participants. A 500 Hz tone was played to mark the end of the neutralization period.

The participant was instructed to press a button through the keypad. After the participant pressed the keypad, we counted 5,000 ms to ensure that there was a rest period of 5000 ms where the fingertip did not touch. After the resting period, a 1,000 Hz tone is played to indicate the start of the thermal stimulation period. The screen displayed a stimulation unit number instructing the participant to touch the thermal pad in the thermal stimulation unit with the desirable thermal stimulation such as 9°C, 15°C, 32°C, and 42°C. The contact between the thermal pad and the fingertip was maintained for a total of 5,000 ms after which a 500 Hz tone was played to mark the end of the stimulation. A question to ask about perceived thermal sensation appeared on the screen instructing the participant to rate their perceived thermal sensation using a five-point Likert scale (very cold, cold, neutral, hot, and very hot). Afterward, a rest period was provided to complete a 30 s duration of the entire trial. It is worth noting that the total trial duration was set to a minimum of 30 s regardless of how quickly the participant touched the stimulation cell or responded to the questions to avoid saturation of the thermal receptor of the participants' fingertip.

### 2.4 Data analysis

As for the EEG data analysis, the EEG data was pre-processed using MATLAB release 2021a (MathWorks, United States) and the EEGLAB toolbox (Delorme and Makeig, 2004). A zero-phase finite impulse response filter with a Hamming window was used for bandpass filtering (0.1–49.5 Hz). The artifact subspace reconstruction method (flat line criterion, 5; high pass filter, 0.25 to 0.75; channel criterion, 0.8; line noise criterion, 4; burst criterion, 10; window criterion, 0.25) (Kothe and Jung, 2016) was applied to remove eye movement and muscle artifacts as well as head movement noise. The EEG signals were then re-referenced using the common average reference method (Lakshmi et al., 2014). The filtered EEG signal was epoched from –5,000 to 7,000 ms corresponding to the thermal stimulation onset as the time when contact was initiated between the fingertip and the thermal pad. –3,000 to –2,000 ms before the thermal onset (resting period) was used as the baseline. After pre-processing, the power spectral densities (PSDs) at each channel at each frequency band were calculated by time/frequency decomposition using the wavelet transform (wavelet cycles entry was 3, 0.8) (Delorme and Makeig, 2004).

The topographies of the delta, theta, alpha, beta, and gamma frequency bands were examined to define regions of interest (ROIs) where the differences in EEG data to the four thermal stimuli were consistently present during the task period. The Jarque-Bera test was used to check whether the PSD associated with the four thermal stimuli followed a normal distribution. One-way ANOVA or Kruskal-Wallis tests were used accordingly. Spectrogram analysis was performed on the ROI where frequency bands showed significant differences. We examined how the PSD was modulated in response to the four thermal stimuli in order to identify frequency bands of interest that encode thermal information.

After determining the ROI and frequency band of interest, a time-course PSD analysis was performed to examine the changes in PSD over time for the four thermal stimuli. After confirming a normal distribution using the Jarque-Bera test, the One-way ANOVA test was used to investigate the PSD differences among the four thermal stimuli. The *p* values were adjusted using the Benjamini and Hochberg false discovery rate (FDR) test. The average of the PSDs during the time period that showed statistically significant differences was considered to examine differences in the PSDs associated with the four thermal stimuli. The One-way ANOVA test was used to examine the differences in PSDs among the four thermal stimuli and *p* values were corrected by the Holm-Bonferroni correction.

The user experience with thermal stimulation was evaluated using self-reporting and EEG data. The participants rated the perceived thermal stimulation using a five-point Likert scale (very cold, cold, neutral, hot, and very hot). The objective of the self-reporting assessment was to cross-validate the EEG data analysis with the self-reported perceptual experience.

## 3 Results

### 3.1 Topography and spectrogram analysis

**Figure 4:**
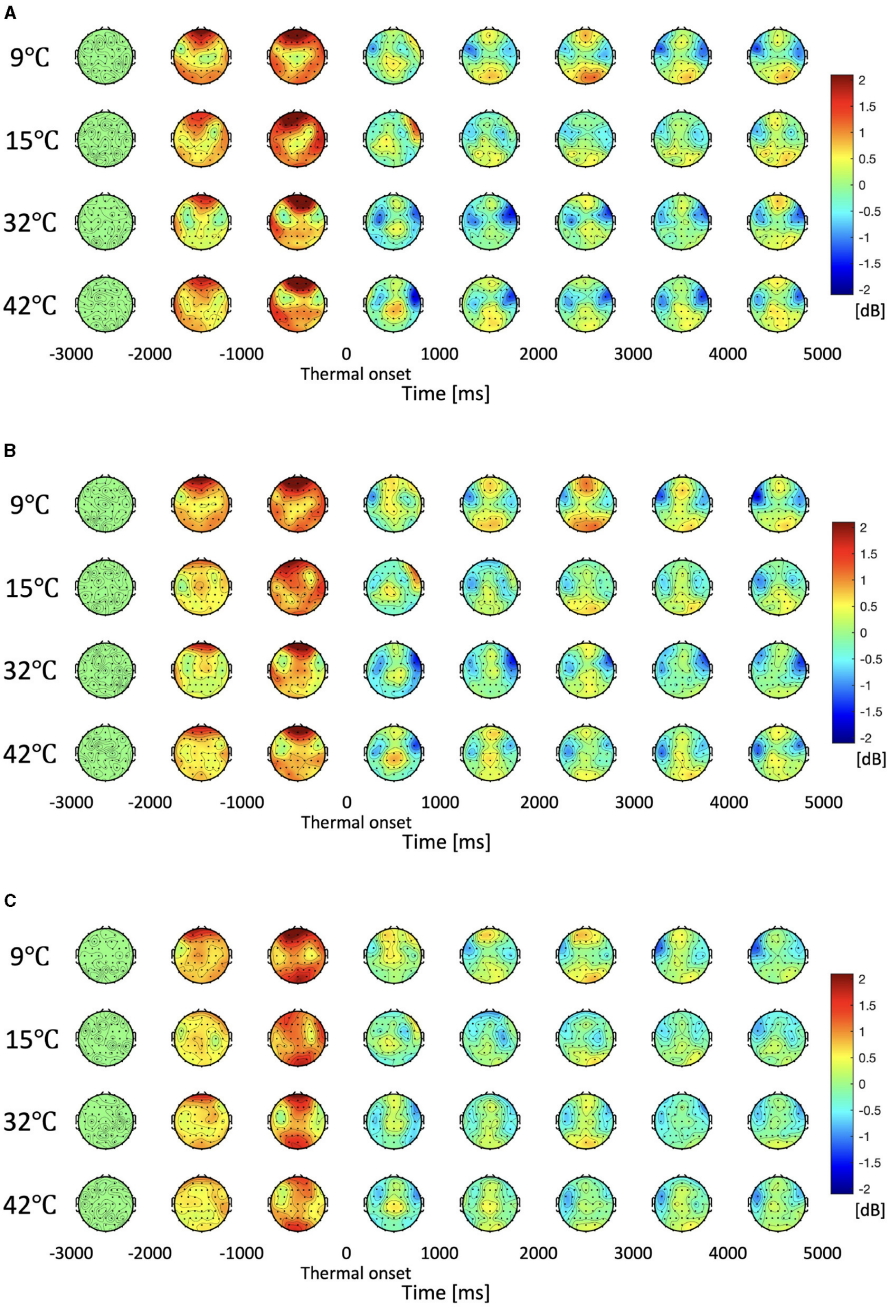
Topographic plotting of delta, theta, and alpha power spectral density. The period from –3,000 ms to –2,000 ms is the baseline. The period from –2,000 to 0 ms (thermal onset) is the finger-reaching to touch the thermal pad. Thermal onset is the moment when the participant's fingertip contacts with the thermal pad. The period of 0 ms to 5,000 ms is the thermal stimulation. The red and blue colors indicate an increase and decrease in power respectively compared to the baseline respectively. **(A)** Distribution of the delta power spectral density. **(B)** Distribution of the theta power spectral density. **(C)** Distribution of the alpha power spectral density.

To determine ROI, we obtained the PSD of delta, theta, alpha, beta, and gamma frequency bands for all electrodes of the EEG data. Figure 4 shows the topography plot for the delta, theta, and alpha PSD at 1,000 ms time intervals. For delta and theta PSD, statistically significant differences in the right frontal area (F6, F8, FC6, FT8, C6, and T8) are observed (One-way ANOVA or Kruskal-Wallis test, Holm-Bonferroni correction, *p* < 0.01). Furthermore, there are significant differences in alpha PSDs in the right frontal area (F6, F8, FC6, and FT8), which is smaller than the region of interest of the delta and theta bands (One-way ANOVA or Kruskal-Wallis test, Holm-Bonferroni correction, *p* < 0.01). In the topography, the period between –3,000 and –2,000 ms was used as the baseline, thus the average value of the period is zero. The –2,000 to 0 ms (thermal onset) interval is the interval where the participant is moving their finger to touch the specified thermal unit. It can be seen in Figure 4 that there is a large PSD activation due to this movement, and after the thermal onset, there is no physical movement, so we can see a stable change. The differences in other brain areas and frequency bands were not statistically significant.

**Figure 5:**
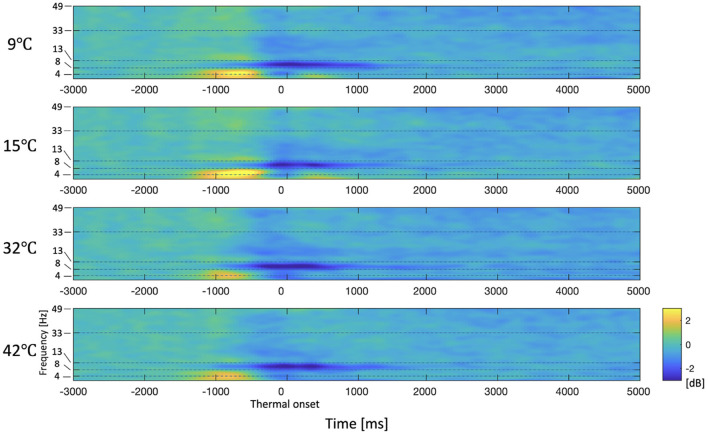
Spectrogram of the average PSD in the right frontal area (F6, F8, FC6, FT8, C6, and T8). The period from –3,000 ms to –2,000 ms is the baseline. The period from –2,000 to 0 ms (thermal onset) is the finger-reaching to touch the thermal pad. Thermal onset is the moment when the participant's fingertip contacts with the thermal pad. The period of 0 ms to 5,000 ms is the thermal stimulation. The yellow and blue colors indicate an increase and decrease in power respectively compared to the baseline respectively.

The spectrogram of the average PSD in the right frontal area (F6, F8, FC6, FT8, C6, and T8), which is the area that exhibits significant differences in the delta and theta frequency bands, is shown in Figure 5. It can be seen that PSDs of the 9°C and 15°C stimuli are generally higher than the baseline while PSDs of the 32°C and 42°C are generally lower than the baseline in low-frequency bands after the thermal onset. There is no significant difference in PSDs between 9°C and 15°C stimuli or between the 32°C and 42°C stimuli. The spectrogram shows that there are no significant differences in high-frequency bands such as beta and gamma bands. Through topography and spectrogram analysis, we are able to determine the frequency bands of interest (delta, theta, and alpha bands).

### 3.2 Time course PSD analysis

**Figure 6:**
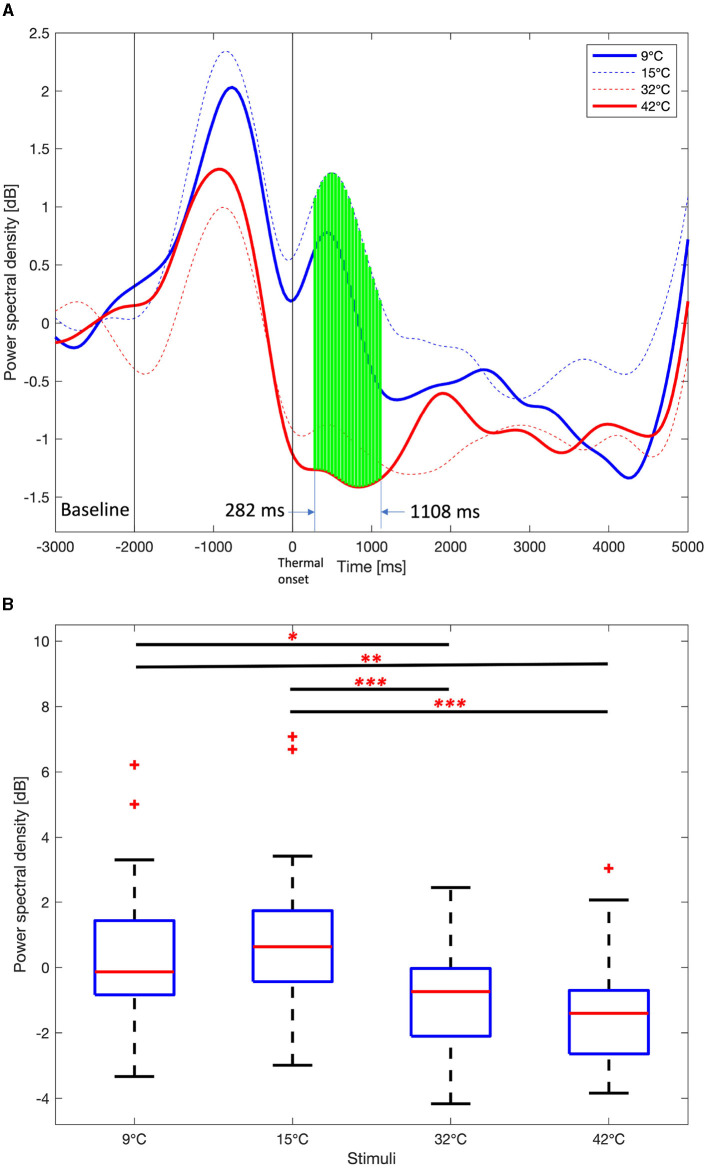
Average Delta power spectral density among 9°C, 15°C, 32°C, and 42°C thermal stimuli in the right frontal area (F6, F8, FC6, FT8, C6, and T8). **(A)** Time course delta power spectral density. One-way ANOVA test or Kruskal-Wallis test, Benjamini and Hochberg false discovery rate correction. Green vertical solid line, *p* < 0.01. **(B)** Mean delta power spectral density from 282 ms to 1,108 ms. One-way ANOVA test, Holm-Bonferroni correction,^*^p < 0.05,^**^p < 0.01,^***^p < 0.001.

The PSDs of the four thermal stimuli over the task period of 5,000 ms was examined. Figure 6A shows the average delta PSD in the right frontal area for the four thermal stimuli over the task period. The interval from –3,000 ms to –2,000 ms was used as the baseline interval in a rest period with no movement after thermal neutralization. After that, –2,000 ms to 0 ms (thermal onset) is the period where there is a movement to touch the stimulation unit, and there are large changes in PSDs. After the thermal onset, PSDs at each time point were examined. The Jarque-Bera test was utilized to verify if the PSD data followed a normal distribution. The one-way ANOVA test was used when all four PSD data were normally distributed. Otherwise, the Kruskal-Wallis test was used. Since the test was performed at each time point, *p* values are corrected by Benjamini and Hochberg FDR. The green vertical solid lines indicate a *p* value < 0.01, indicating a statistically significant difference in four stimuli at a particular point in time. It was observed that PSDs of 9°C and 15°C are higher than the PSDs of 32°C and 42°C from 282 ms to 1,108 ms after the thermal onset. Therefore, the average of PSDs in this time interval was considered to examine the differences between the four stimuli. The PSDs for the 9°C and 15°C stimuli peak at an earlier time than the 32°C and 42°C stimuli.

Figure 6B shows boxplots of the average delta PSDs for the four thermal stimuli from 282 ms to 1,108 ms after the thermal onset. The Jarque-Bera test confirmed that all four PSD data follow a normal distribution, thus the one-way ANOVA test was performed and *p* values were corrected using the Holm-Bonferroni correction. There are no significant differences in the average theta PSD over the task period between 9°C and 15°C and between 32°C and 42 °C. However, there are significant differences in the average delta PSD between 9°C/15°C and 32°C/42°C (One-way ANOVA, Holm-Bonferroni correction, *p* < 0.05).

**Figure 7:**
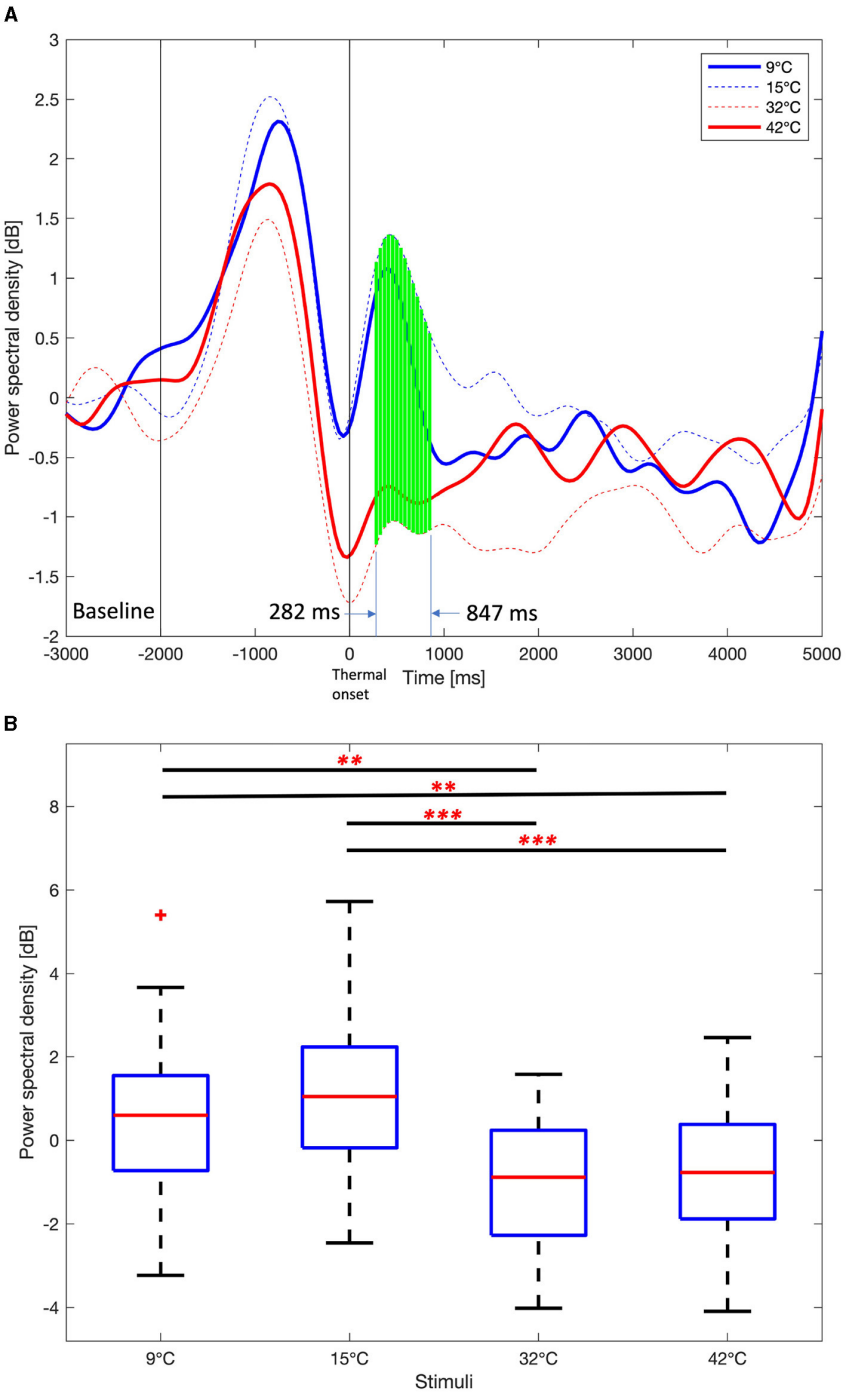
Average Theta power spectral density among 9°C, 15°C, 32°C, and 42°C thermal stimuli in the right frontal area (F6, F8, FC6, FT8, C6, and T8). **(A)** Time course theta power spectral density. One-way ANOVA or Kruskal-Wallis test, Benjamini, and Hochberg false discovery rate correction. Green vertical solid line, *p* < 0.01. **(B)** Mean theta power spectral density from 282 ms to 847 ms. One-way ANOVA test, Holm-Bonferroni correction,^**^p < 0.01,^***^p < 0.001.

Time course average theta PSDs in the right frontal area in Figure 7A also show a similar pattern to time course delta PSD. However, the time interval of statistically significant differences (One-way ANOVA test or Kruskal-Wallis test, Benjamini and Hochberg FDR correction, *p* < 0.01) in theta PSDs was narrower than the interval of differences in delta PSDs. The interval with a statistically significant difference in theta PSDs is 282 ms to 847 ms, and the interval with a statistically significant difference in delta PSDs is 282 ms to 1,108 ms. Similar to delta PSDs, the peak of theta PSDs after the thermal onset was earlier for the 9°C and 15°C stimuli than for the 32°C and 42°C stimuli. The difference in mean theta PSDs between the 282 ms and 847 ms are shown in Figure 7B. The PSDs for the 9°C and 15°C stimuli are statistically higher than the 32°C and 42°C stimuli (One-way ANOVA test, Holm-Bonferroni correction, *p* < 0.01). This is a similar result to the delta PSDs.

**Figure 8:**
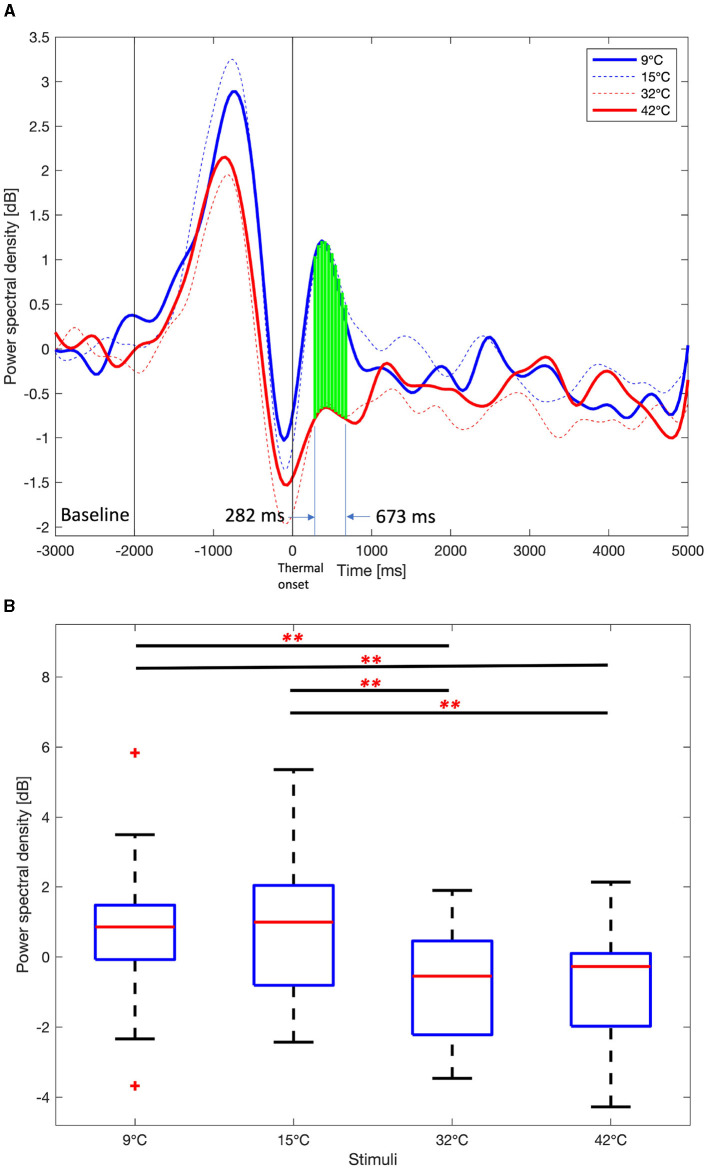
Average Alpha power spectral density among 9°C, 15°C, 32°C, and 42°C thermal stimuli in the right frontal area (F6, F8, FC6, and FT8). **(A)** Time course alpha power spectral density. One-way ANOVA or Kruskal-Wallis test, Benjamini and Hochberg false discovery rate correction. Green vertical dashed line, *p* < 0.05. Green vertical solid line, *p* < 0.01. **(B)** Mean alpha power spectral density from 282 ms to 673 ms. One-way ANOVA test, Holm-Bonferroni correction, ^**^p < 0.01.

Figure 8 shows the time course average alpha PSDs in the right frontal area and boxplot for the mean alpha PSDs in the interval that showed significant differences in the thermal stimuli (One-way ANOVA test or Kruskal-Wallis test, Benjamini and Hochberg FDR correction, *p* < 0.01). The alpha PSDs results are similar to those obtained for the delta and theta PSDs. However, the ROIs for alpha PSDs are F6, F8, FC6, and FT8, which are smaller than F6, F8, FC6, FT8, C6, and T8 for delta and theta. The differences in alpha PSDs between the lower thermal stimuli (9 and 15°*C*) and the higher thermal stimuli (32 and 42°*C*) compared to the neutralizing temperature (23°*C*) are also smaller than the difference between delta and theta PSDs.

### 3.3 Subjective evaluation

**Figure 9:**
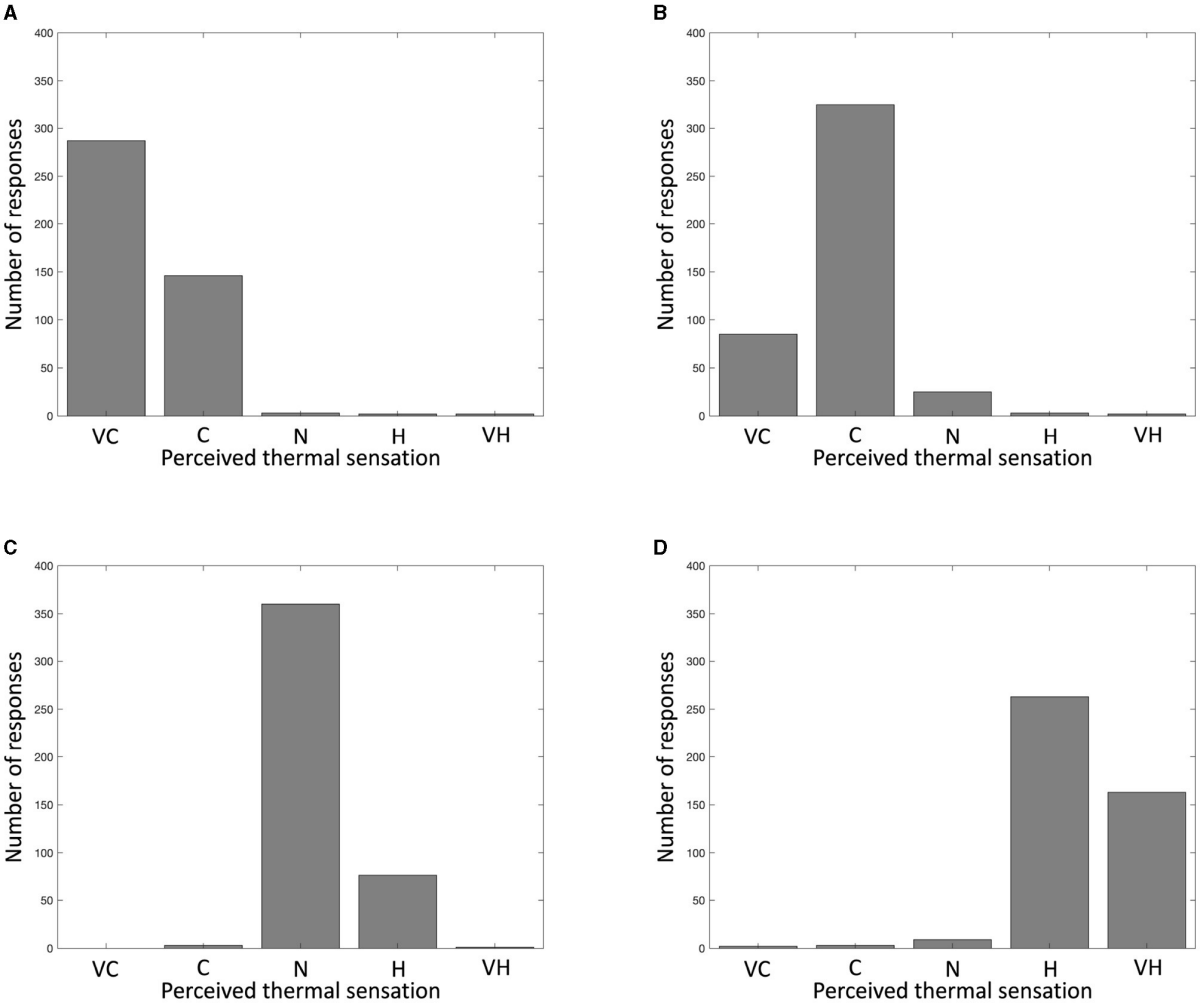
Histograms of participants' perceived thermal sensation. VC, C, N, H, and VH indicate very cold, cold, neutral, hot, and very hot respectively. **(A)** 9°C thermal stimulation. **(B)** 15°C thermal stimulation. **(C)** 32°C thermal stimulation. **(D)** 42°C thermal stimulation.

The subjective evaluation aims to assess the correlation between the physical thermal stimuli and their respective perceptual experiences. Before providing the thermal stimulation, participants experienced the neutralization period with 23°C thermal stimulation to minimize any bias or masking effects. The results are shown in Figure 9. For the 9°C thermal stimulation, about two-thirds of the participants perceived it as very cold while about one-third of the participants perceived it as cold (Figure 9A). As shown in Figure 9B, the 15°C thermal stimulation is dominantly perceived as cold with a few participants perceiving it as very cold or neutral. For the 32°C thermal stimulation, the dominant response is neutral, with some participants perceiving it as hot as seen in Figure 9C. For the 42°C thermal stimulation, participants perceived it more often as hot than very hot, as clearly demonstrated in Figure 9D. Even if the participants felt the same thermal stimulation, their perceived thermal sensations were different.

## 4 Discussion

The PSDs in the delta, theta, and alpha bands in the 9°C and 15°C thermal stimuli were significantly higher than the PDSs in the 32°C and 42°C thermal stimuli in the right frontal area. There was no significant difference in PSDs between 9°C and 15°C thermal stimuli or between 32°C and 42°C thermal stimuli. However, this is different from previous studies that have investigated EEG responses to thermal stimuli. In a study by Tayeb et al. (2022), participants were asked to hold balls that were very cold, cold, warm, hot, and very hot, and their EEGs were examined. Their results showed that bilateral temporal alpha suppression was observed at very cold (10–14.99°C) and very hot (40.99–44.99°C) stimuli. However, this was not seen in the present experiment. In Tayeb et al. (2022)'s experiment, the balls were held in the palm of the hand, thus the contact location and area for thermal stimulation are different. Also, participants were exposed to the stimuli for 30 s, which may have contributed to multiple confounding responses. Also, the sample size was limited to 10 trials for each stimulus with only 3 participants. The study by Wang et al. (2021) considered high temperatures of 33–41°C and found an increase in alpha and beta activation. The location of the thermal stimulus is on the head, which is different from the fingertip. Rather than examining the difference in PSD of each frequency band relative to the baseline, this experiment investigated the temperature-dependent difference in PSD of each frequency band with baseline-corrected PSD. Han and Chun (2021)'s study investigated the change in ambient temperature and the results showed that beta and gamma power increases and theta power decreases with ambient temperature changes. The significance of this study is that it is the first in which the thermal stimulation is applied at the fingertip after active hand movement, which is a common way to thermally explore the environment in daily life.

More interestingly, the changes in the delta, theta, and alpha PSDs in the right frontal area happened at an early stage (from 282 ms to max. 1,108 ms). For vibrotactile sensation, ERD/ERS and ERP responses in the somatosensory area occur within one-second (Park et al., 2021), and EEG responses to friction on a surface are also observed within one-second (Park et al., 2019). Differences in EEG responses for higher-order cognitive functions such as satisfaction with a stimulus also may also be experienced within two seconds (Park et al., 2018). This study shows that the response time to thermal stimulation, similarly to other haptic stimulation, occur within approximately one second. The PSDs for the 9°C and 15°C stimuli are below the ambient/neutralization temperature of 23°C and show a peak within 1 s. 32°C and 42°C PSDs are above the ambient/neutralization temperature of 23°*C*, and the peak occurs after 1 s. This might be attributed to the fact that the thermal receptors that detect hot temperatures are slower to respond that thermal receptors that detect cold temperatures (Hensel, 1974). The results are likely due to differences in thermal receptors, and further research is needed to understand why differences were seen in low-frequency bands such as delta, theta, and alpha in the right frontal area, but not in the beta and gamma bands.

In addition, vibrotactile sensation showed different EEG responses depending on the intensity of stimuli (Park et al., 2021), however, the results from thermal stimuli do not show significant differences between 9°C and 15°C or between 32°C and 42°C, which was unexpected. The reason for these results can be found in the results of the participants' perceived thermal sensation. Participants' self-reporting clearly indicated individual differences in perception of thermal stimulation. Each participant may have different mental or physical characteristics as well as different temperature standards for (very) cold and (very) hot depending on their personal experiences (Schweiker et al., 2018). This may affect participants' perceived thermal sensation. It is also interesting to note that even for the same temperature stimulus, the perceived thermal sensation varies depending on the participant's gyroscopic environment and background. From the participants' responses, there is no clear distinction in the perceived thermal sensation between 9°C and 15°C or between 32°C and 42°*C*. This explains the lack of significant difference in the PSDs between the 9°C and 15°C thermal stimuli and between 32°C and 42°C thermal stimuli. Further research using machine learning models can clarify this. There is one study that uses deep learning to classify EEG responses based on haptic properties (Alsuradi et al., 2023). Nonetheless, it is expected to see differences at extreme cold with temperatures lower than 9°C or extreme hot with temperatures higher than 42°C due to pain or discomfort.

The neural correlates of thermal stimulation were investigated using fMRI (Oi et al., 2017). At low thermal stimulation, there are significant activations in the left dorsal posterior insula, putamen, amygdala, and bilateral retrosplenial cortices. There are also fMRI studies that show that applying painful thermal stimulation to both hands can elicit significant activity in a broad network of brain regions, including the insula, inferior frontal gyrus, cingulate gyrus, secondary somatosensory cortex, cerebellum, and medial frontal gyrus (Brooks et al., 2002). However, the activation is in subcortical regions, so a direct comparison with the EEG responses in this study is not possible. There are also studies that have shown increased activation in the motor-related cortex with alternate hot and cold thermal stimulation (Chen et al., 2019). Further research is needed to determine whether these studies correlate with fMRI studies showing neural changes in the deep brain and EEG changes in cortical regions of the brain in response to thermal sensation.

The experimental protocol also involved auditory and visual cues. Thus, the potentials evoked by auditory (1,000 Hz and 500 Hz tones) and visual (thermal unit numbers) cues can occur. However, in this study, the auditory and visual cues are applied equally to the four thermal stimulation conditions. There was also motor movement before the thermal onset. Thus, an alpha ERD is expected in the contralateral sensorimotor cortex. However, this is difficult to see in the topography. This is probably due to the fact that the onset is not synchronized by movement, but is based on the moment when the fingertip makes contact with the thermal pad. In the present study, an experiment was designed to observe the EEG response to thermal sensation rather than the known EEG response to motor movement. Furthermore, the motor movement is very similar for all four conditions. We found significant differences in the EEG responses across the four conditions, making it unlikely that this is an artifact of motor movement.

We can see the PSD differences in temperature even before the thermal onset. While these are not statistically significant differences, we can speculate on a few reasons why this might be happening. It is possible that the change in power is due to the movement of the participant's hand in stretching and placing the fingers on the thermal pad. However, this movement is the same for all four temperatures. We believe that there are two reasons why there appears to be a difference before the onset. First, we used the 3-cycle wavelet transform, which is generally recommended for wavelet transforms for the time-frequency analysis (Roach and Mathalon, 2008). For low frequencies, the time period corresponding to three cycles is longer and tends to be smoothed on the time scale. Another reason is that the numbering of the thermal units representing the thermal stimuli in the counterbalance order was not perfectly randomized. This was chosen to allow sufficient time to change the temperature of the thermal unit without overloading the hardware. As a result, when participants saw the numbering of the thermal units, they may have had an expected temperature in mind, which could have led to a change in PSD associated with prediction processes.

Although the current study investigated neural correlates as a basis for quantitatively evaluating the experience of thermal stimulation, there are some limitations that require further research to validate and expand upon. The participant sample had a limited age range, with the majority falling between 18–25 years old. There were also fewer female participants. The generalizability of the findings to different age groups and genders may be limited. The results also did not account for external factors that could influence thermal perception, such as individual differences in cold or heat tolerance, adaptation, or variations in clothing or environmental conditions. Conducting the study in a different cultural context may introduce cross-cultural variations in thermal perception and neural responses. Furthermore, providing thermal stimulation at different body parts, such as the arm, face, and calf may result in different neural correlates. We collected the perceived thermal sensation using the 5-Likert scale, however, the analog scale is very helpful to avoid bias and to accurately capture the perceived thermal sensation. Finally, the haptic properties of the thermal stimulation surface (stiffness, texture, wetness) could modulate thermal perception (Ho et al., 2019) and should therefore be investigated.

## 5 Conclusions

This study aimed to investigate the neural correlates associated with various levels of thermal stimulation using EEG brain imaging. The thermal setup was developed as part of this study to provide thermal stimulation at the fingertip at four temperature levels while brain response was recorded using an EEG system. The findings of the study have important implications for the development of human-computer interaction systems that incorporate thermal feedback. The topography and spectrogram analysis identified specific regions of interest (the right frontal area) and frequency bands (delta, theta, and alpha) that encode thermal information. The time-course PSD analysis showed significant differences at the early stage after the thermal onset in the four thermal stimuli, indicating distinct neural responses to different levels of thermal stimulation. The study contributes towards developing a quantitative, real-time, and non-intrusive system for evaluating the user experience with thermal feedback.

## Data availability statement

The raw data supporting the conclusions of this article will be made available by the authors, without undue reservation.

## Ethics statement

The studies involving humans were approved by IRB: #HRPP-2020-80/New York University Abu Dhabi. The studies were conducted in accordance with the local legislation and institutional requirements. The participants provided their written informed consent to participate in this study.

## Author contributions

WP: Conceptualization, Formal analysis, Funding acquisition, Investigation, Writing—original draft, Writing—review & editing. GK: Investigation, Methodology, Software, Writing—review & editing. MJ: Investigation, Methodology, Software, Writing—review & editing. ME: Conceptualization, Funding acquisition, Project administration, Supervision, Writing—review & editing.
